# A qualitative investigation of the experience of participation in Mindfulness‐based Intervention for IVF‐ET (MBII) with Chinese women undergoing first IVF‐ET

**DOI:** 10.1002/nop2.232

**Published:** 2018-12-18

**Authors:** Jing Li, Hong Luo, Ling Long

**Affiliations:** ^1^ Western Research Base of Sociology Chongqing Technology and Business University Chongqing China; ^2^ Institute of Reproductive and Genetic Health Center for Women and Children Chongqing China; ^3^ Reproductive Medical Center, Southwest Hospital Third Military Medical University Chongqing China

**Keywords:** fertility quality of life, in vitro fertilization Embryo Transfer, mindfulness‐based intervention, qualitative research

## Abstract

**Aim:**

To explore what the women experience during their first IVF‐ET treatment while participating a mindfulness‐based intervention for IVF‐ET (MBII), and how they use it to enhance their infertility‐related quality of life (QoL).

**Design:**

Qualitative exploratory design.

**Methods:**

As part of a larger multimethod study, this study shares the description from 38 IVF‐ET women. We conducted semi‐structured interviews and collected daily practice diary. Each interview was conducted one‐on‐one within one week after MBII programme. Data were analysed using inductive thematic analysis.

**Results:**

Four primary themes described participants’ perceptions of how the programme benefitted IVF‐ET treatment and daily lives: improved infertility‐related QoL, enhanced awareness, increased acceptance and regained control over life. Additionally, enhanced awareness, regained control over life and increased acceptance may be implicated in the effectiveness of MBII on the infertility‐related QoL. Clinicians and nurses working with women undergoing first IVF‐ET have another tool to recommend to the patients.

## 1 INTRODUCTION

Infertility is defined by the International Committee for Monitoring Assisted Reproductive Technology (ICMART) and the World Health Organization as the “failure to achieve a pregnancy after 12 months or more of regular unprotected sexual intercourse” (Zegers‐Hochschild et al., 2009). It can also be considered as a social and emotional condition described as a low‐control stressor in which the couple is challenged with the unfulfilled goal of parenthood (Greil, 1997). Dealing with infertility is frequently perceived as a physically and psychologically challenging experience. Furthermore, infertile women often use in vitro fertilization Embryo Transfer (IVF‐ET) treatment to realize their wish to have children. However, an IVF‐ET treatment is a painful and stressful process including the daily injections, blood samples, ultra‐sound scan, which might bring along some negative effects on a woman's quality of life (QoL) profoundly and enduringly (Kaliarnta, Nihlén‐Fahlquist, & Roeser, 2011; Xiaoli et al., 2016). Poor QoL in turn often leads to undesirable consequences such as fewer treatment cycles and a lower mean number of live births (Sejbaek, Hageman, Pinborg, Hougaard, & Schmidt, 2013). Therefore, QoL of infertile women is one of the most important issues to be addressed in infertility counselling (Ferreira, Vicente, Duarte, & Chaves, 2015; Haica, 2013). Fertility quality of life (FertiQoL) is specifically designed for infertile patients to assess their QoL, including two main modules, the Core FertiQoL module and the optional Treatment module (Boivin, Takefman, & Braverman, 2011). Core FertiQoL evaluates the impact of infertility on patients’ emotions, physical health, cognition, behaviour, partnership and social aspects. Whereas, Treatment FertiQoL includes environment and tolerability FertiQoL. FertiQoL is condition‐specific and aims to measure QoL in all people experiencing fertility problems, and a reliable measure of the impact of infertility on QoL (Aarts et al., 2011). The FertiQoL tool has been translated into 20 different languages.

### 1.1 Background

Numerous studies have proven effective psychological interventions could reduce anxiety, depression, uncertainty and other psychological issues, whilst improving fertility‐specific QoL and pregnancy rates among infertile women (Chan, Ng, Chan, & Chan, 2006; Domar et al., 2000; Hosaka, Matsubayashi, Sugiyama, Izumi, & Makino, 2002; Kim et al., 2014; Noorbala et al., 2008; Oron et al., 2015). Mindfulness meditation is one example of complementary therapy that has been applied in behavioural medicine (Baer, 2003; Bishop, 2002). Mindfulness is commonly and operationally defined as the quality of consciousness or awareness that arises through intentionally attending to present moment experience in a non‐judgemental an accepting way (Kabat‐Zinn, 1994). Mindfulness‐based interventions (MBIs) integrate the essence of traditional mindfulness practice with contemporary psychological practice, in order to improve psychological functions and well‐being (Gu, Strauss, Bond, & Cavanagh, 2015). A growing body of robust evidence has demonstrated that MBIs are effective in improving QoL in comparison with control conditions among some populations, such as breast and prostate cancer patients (Witek‐Janusek et al., 2008), people with generalized anxiety disorder(Morgan, Graham, Hayes‐Skelton, Orsillo, & Roemer, 2014), and recurrently depressed patients(Godfrin & Van Heeringen, 2010). Moreover, MBIs significantly ameliorated the quantitatively measured outcomes among infertile women, such as infertility stress (Peterson & Eifert, 2011), depressive symptoms, internal and external shame, entrapment, defeat and self‐efficacy to deal with infertility (Galhardo, Cunha, & Pinto‐Gouveia, 2013). Our research team conducted an evaluation of an adapted mindfulness‐based intervention for IVF (MBII) aimed specifically at improving the FertiQoL and related psychological constructs of women receiving IVF‐ET. The study demonstrated that MBII improved mindfulness, self‐compassion, emotion regulation and coping strategies, thus increasing FertiQoL and pregnancy rates.

However, these quantitative findings did not describe the means by which participants used mindfulness to improve infertility‐related QoL or other effects of meditation. There are not first‐person descriptions in the literature that describes what the women experience during their first IVF‐ET treatment while learning mindfulness meditation, and how they use it to enhance infertility‐related QoL. A purely quantitative approach to understanding the effects of mindfulness training is limited, whereas qualitative approaches may add greater insight into psychological mechanisms and characteristics associated with mindfulness than a self‐report mindfulness scale alone (Grossman, 2008), and help generate hypotheses for quantitative research methods (Morone, Lynch, Greco, Tindle, & Weiner, 2008).

### 1.2 Aim

Our objective was to explore what the women experience during their first IVF‐ET treatment while participating the MBII programme, and how they use it to enhance their infertility‐related QoL.

## 2 METHODS

### 2.1 Study Context and interventions

The present study was embedded in a non‐randomized controlled trial (Li, Long, Liu, He, & Li, 2016) (from November 2013–November 2014) in which 50 women were enrolled in the control group, and 58 in the intervention group. The study was conducted in the fertility medical centre in Southwest Hospital in China. Written informed consent was obtained from all individual participants included in the study. The study protocol was approved by the Ethics Committee of Third Military Medical University prior to participant recruitment. The work described in this paper was carried out in accordance with the Code of Ethics of the World Medical Association's Declaration of Helsinki.

The MBII was based on the Mindfulness‐based Stress Reduction (MBSR，Kabat‐Zinn, 2013; Stahl & Goldstein, 2010), Mindfulness‐based Cognitive Therapy (MBCT (Segal, Williams, & Teasdale, 2002), Mindfulness‐based Childbirth and Parenting (MBCP (Bardacke, 2012) and Acceptance and Commitment Therapy (ACT; Hayes, Strosahl, & Wilson, 1999). Participants met once a week for a 2–2.5‐hr session for 6 weeks. In addition to in‐class mindfulness exercises, the women were encouraged to engage in home mindfulness practices. A total of six groups with 6‐8 women per group completed the programme within one year. Sessions were led by the first author who is an experienced clinical psychologist with training in mindfulness‐based approaches, and psychological interventions for infertile patients. The techniques used were as follows: body scan, sitting meditation, mindful yoga, walking meditation, loving‐kindness meditation, mindfulness anxiety meditation, raisin‐mindful eating and mindfulness pain meditation. Meditation practice was supported between sessions through audio‐recorded instructions and handouts outlining ways to apply mindfulness skills to daily lives. The study protocol also included homework of daily meditation (Li et al., 2016).

### 2.2 Sample

Participants were invited to this qualitative study if they: (a) had attended all six sessions; (b) completed a daily diary about their experience completely; (c) had been semi‐structured interviewed; and (d) had no language obstacles and agreed to participate in this study. A sample of 38 women (70.7% response) in the intervention group (*N* = 58) in our quantitative study was interviewed. Recruitment to the study was based on the inclusion criteria. Of the 58 participants allocated to the MBII group, 48 were interviewed (10 did not attend all of six sessions and/or not filled out a daily diary about their experience completely). The final 38 interviews were selected for analysis based on the information saturation principle. There were no significant differences in demographic, clinical and study variables between 38 participants in this qualitative study and 20 not in the study. Of the 38 participants, the mean age was 30.66 years (between 22–45 years), 42% attended college or had a tertiary education, 32% were unemployed, 59% had a female factor cause for their infertility, the mean infertility duration was 4.7 years (range 1–14) and the mean infertility treatment duration was 2.3 years (range 1–8).

### 2.3 Data collection

Since diary entries and interview can reveal a rich depth of experience, we conducted semi‐structured interviews and collected daily practice diary.

Each semi‐structured interview, which took approximately 30 min, was conducted one‐on‐one within one week after the last session in each group at the reproductive medical centre in Southwest Hospital. Based upon the FertiQoL survey in our quantitative study, seven overarching questions inquiring about the women's experiences in relation to the topics within the FertiQoL guided the interviews (Table 1). The focus was the women's experience of MBII as guided by FertiQoL topics of emotions, mind‐body, marital relationships, social relationships, satisfaction with medical quality and treatment tolerability. This interview approach permitted discussion and allowed for data to enter interview that was not directly sought. All interviews were tape‐recorded with consent and transcribed verbatim by the interviewer.

**Table 1:** Interview topics and main questions

Topic	Questions
Emotions state	Please talk about your infertility and IVF‐related emotional state following the mindfulness course
Mind‐body state	Please talk about your infertility and IVF‐ related physical and mental state following the mindfulness course
Marital relationship	Please talk about your marital relationship following the mindfulness course
social relationship	Please talk about your social relationship following the mindfulness course
Perceived satisfaction with the medical staff and received medical services	Please talk about your experiences of your received medical services and the interaction with medical staff following the mindfulness course
Tolerability of IVF treatment	Please talk about your tolerability for IVF‐related experience following the mindfulness course
Total comment on the program	What do you think your participation in the course has offered you?

Note

IVF: in vitro fertilization.

Additionally, the study protocol also covered homework of daily meditation, including practicing mindfulness techniques and completing daily practice diary. After completing mindfulness practice at home each day, participants were required to write down relevant information in the practice log. The headings of the daily practice diary included “Date,” “Time,” “Place,” “Thoughts, emotions and body feelings during the practice,” and “The current stage of your IVF‐ET treatment.” The women's daily practice diaries were collected weekly and returned to the women each week following the transcription. One difficulty with using handwritten diaries can be that entries are sometimes illegible. One investigator transcribed the diaries and another investigator double‐checked each transcription and reviewed difficult‐to‐read handwritten notes. Ultimately only 1% of diary entries were illegible to coders. The participants’ quotations were assigned a number that consisted of a “P” (for participant), the participant's randomization number, the page and the line numbers of the quotation within each transcript (e.g. P1/1/1–2 for Participant 1, Pages 1, Lines 1–2).

### 2.4 Data analysis

The diary entries and interview transcripts were analysed using thematic analysis for its flexibility and potential to generate unanticipated insights (Clarke & Braun, 2013). The analysis followed thematic analytic procedures: becoming familiar with data involving transcription and reflective reading, generating initial codes, searching for themes, reviewing and refining themes, identifying coherent patterns, defining and naming themes and producing the report (Braun & Clarke, 2006). In addition, some themes emerged from the analysis of the interviews, some from diaries, and some from both interviews and diaries.

NVivo qualitative analysis software was used to code transcripts. A peer‐review process took place as a validation strategy to control for author bias in the interpretation of themes (Himelstein, Hastings, Shapiro, & Heery, 2012). A qualified research colleague neutral to this study received full transcripts of all interviews and diaries, and reviewed the codes and themes completely independently of the primary investigator’ s participation. This peer reviewer reviewed each code against each meaning unit and quote from the transcripts. After the primary investigator and neutral colleague met to compare and contrast coded themes, member checking was used to validate the interpretation of the data.

## 3 RESULTS

Following a thematic analysis of the data, four major themes were identified which captured patients’ experiences of mindfulness in relation to their infertility, IVF treatment and daily lives. These included improved infertility‐related QoL, enhanced awareness, regained control of lives and increased acceptance (Table 2).

**Table 2:** Summary of over‐arching themes and themes

Over‐arching themes	Themes
Improved infertility‐related QoL	*Infertility‐related emotions:* being calmer, more relaxed, at peace.
	*Infertility‐related Mind‐body state: *alleviating physical discomfort and mood fluctuations during IVF treatment, improved sleep.
	*Relationa*l QoL: enhanced closeness with partner.
	*Social relationship:* cherishing social connections more, less isolation and inferiority, more social inclusion.
	*Perception on the treatment environment: *be more satisfied with the medical staff and received medical services.
	*Tolerability of IVF‐ET treatment:* accepting and going with the IVF.
Enhanced awareness	*Increased awareness of their bodies, emotions and thoughts*
	*Living in the moment more often*
	*Greater concentration*
Regained control over life	*Awakening both body and mind*
	*Increasing self‐efficacy to deal with infertility and IVF‐ET*
Increased acceptance	*Destigamanization*
	*Infertility and IVF‐treatment related thoughts and feelings objectified*
	*More kind and compassionate to themselves and others*

Note

IVF: in vitro fertilization.

### 3.1 Improved infertility‐related QoL

This over‐arching theme described participants’ perceptions and evaluations of their infertility‐related QoL following the MBII course, including the following six sub‐themes.

#### 3.1.1 Improving their infertility‐related emotions

Post‐MBII programme, all 38 women were aware of inner calm that helped them feel better and think clearly. One participant said as follows:

When I think about my infertility and IVF treatment, I am so sad and helpless. However, focusing my attention on the breathing in and out, I can return to calmness and relaxation. Breathing is just like my soul harbor. I am less irritable and impatient than before. (P1/1/12–14)

The mindfulness practice offered a way by which participants monitored and controlled their own arousal and could cope with their problems with greater equanimity:

When a strong emotion arises, I try to take a moment to mindfully return to my body to discover physical sensations associated with that emotion. I was used to being nervous when the doctors checked or operated on me. But now I can detect my physical response such as trembling hands, and then begin to practice mindfulness breathing or body scan. Soon, I can calm myself down, because I know that the emotion will be coming and going, just like the clouds in the sky. (P5/8/24–28)

#### 3.1.2 Improving their infertility‐related mind‐body state

Twenty‐nine women said that these practices helped them “deal with physical pain and other discomforts” (P36/75/13–14), alleviating fatigue and emotion fluctuation during treatment. Mindfulness techniques can directly help them lessen surgery pain such as the hysteroscopic operation‐related pain. One participant gave a concrete description of her experience in the interview.

The mindfulness techniques reduce my physical pain during the hysteroscopic operation. It was too painful. At that time, I redirected my attention on the breath, feeling my body arising with breathing in, and falling with breathing out. Gradually I became calm, suffering less and feeling better. I can not stop the pain, but I can ride on the waves of sensations, letting them be, letting them go. (P24/52/15–16)

Additionally, 35 women described varying sleep improvements in the diaries:

I have been suffering from insomnia because of my infertility and IVF. During the MBII, I often use body scan to help me fall asleep again. Now sleep comes very easily. Sleep latency is reduced and more refreshing. Mindfulness makes a huge difference in my life. (P20/35/21–23)

#### 3.1.3 Improving their relational QoL

Post‐MBII programme, 29 participants said that MBII had enhanced closeness with partner, increasing their marital satisfaction.

My husband supports me in this program. I share what I learned in the course with him. We can communicate a lot on infertility, IVF, and other things openly. A happy marriage is very important for me. I am more satisfied with my husband and marriage, and we are closer to each other now. (P1/1/23–24)

#### 3.1.4 Improving their social relationship

Post‐MBII, 30 participants reported in the interview and diaries that they cherished social connections more, feeling less isolation and inferiority, thus more satisfaction with their social relationship.

I no longer withdraw myself from the social circle. I am more “present” with my friends, having less anxious and inferiority in some situations or activities. I enjoy being with my friends, whatever it is climbing a mountain together or walking in the sunshine with laughter. How happy I am, just like facing the sea with spring blossoms. We talk about worries with each other, making me to relax. I began to tell my friends about my IVF‐ET, which is a relief. Cherish my friends! (P9/15/24–28)

#### 3.1.5 Improving their perception on the treatment environment

Post‐MBII, 25 participants experienced changes in the relationship with their thoughts, in that they became more aware of them, as well as detaching from them, thereby observing them from a new perspective. A lot of participants reported more satisfaction with the interaction with medical staff and medical service in the diaries and interview:

After I attend to this course, I have changed my attitude toward the medical staff. I often distract my attention from the anxiety about treatment, and focus on the people around me. Watching the busy nurses, I know that nobody lives easily, so I have more empathy for them. (P20/36/27–28)

Another participant said as follows:

This program has taught me that there is a space between my IVF‐ET stressor and response, and I can choose mindfulness response in that space, such as actively seeking adaptive coping strategies. Thus I can control my behaviors in difficulty situations such as encountering unpleasant medical services. When nurses give me a cold shoulder, or make mistakes, I will tell myself that sometimes nurses are upset because of dealing with so many patients in each day. So that really helped me a lot. (P35/73/14–15)

#### 3.1.6 Improving their tolerability of IVF‐ET treatment

Post‐MBII, twenty‐eight women reported that they were happy to have these mindful tools to help them to reduce treatment stress and increase treatment tolerability in the diaries:

I always worry about my follicular growth and endometrial pattern. Sometimes, I think of dead fetuses, making me panic. However, during sitting meditation, I am in calmness and stillness because I learn that thoughts will come and go, just as clouds do in the sky. Moreover, body scan is useful to soothe my uterus. I also regard mindful breathing as a reward to me. I divide unpleasant experience into thoughts, emotions and body sensation. Then I can respond it in a creative way, and not perceive it as overwhelming. Therefore, I have greater tolerance for my IVF. (P20/36/5–8)

### 3.2 Enhanced awareness

This theme describes that participants increased awareness of their bodies, emotions and thoughts, and could live their moments fully and completely, as well as were able to pay more attention and sustain that attention when stressful invading thoughts, mind wandering and ruminations come to mind. This theme included the following sub‐themes.

#### 3.2.1 Increasing awareness of their bodies, emotions and thoughts

Post‐MBII, one of the major changes all 38 participants reported in the interviews and diaries was an increased awareness of their bodies, and how bodily sensations could be related to thinking and emotions.

For a long time, my infertility and IVF‐ET have dominated my life; I “lived a little distance from my body”. Now, I really know, oh, I have a body! I can feel my body now! I can listen to my body and mind! Awareness is the best gift from mindfulness meditation for me. (P22/47/23–25)

#### 3.2.2 Living in the moment more often

Thirty‐one participants said that they could live their moments fully and completely. Participants shared how they were better able to enjoy little things in life:

Now even listening to each drop of rain becomes so enjoyable. I enjoy the richness of life, such as smelling the fresh air, feeling the wind in my face and the sun's warmth on my skin. I can taste plain boiled water silently for a long time, making me comfortable! Mindfulness teaches you to be here now. (P1/1/29–30)

In addition, paying attention to sensations whether they were walking or following their breath helped them cope with negative emotion and thoughts. As one person said:

Just living in the moment, not the past or future can awaken my inner resources for calmness, confidence, and healing. Once I concentrate on the things that I am doing, whether I am walking or eating, or following my breath, I can become calmer, with less complains and anxiety about my IVF‐ET (P4/6/13–14)

#### 3.2.3 Greater concentration

Twenty‐six participants reported that they were able to pay more attention and sustain that attention when stressful invading thoughts, mind wandering and ruminations come to mind. They also were better aware of and able to manage distractions by bringing their concentration back to their breath. One participant said in the interview.

After practicing mindfulness meditation, I found that I could focus on the things that I was doing, such as reading. Although still being distracted, I can return to the moment right now, thus improving work efficiency. (P3/22–23)

### 3.3 Regained control over life

Out of control is a common state for infertile patients. Their lives became so dominated by the infertility experience that they stopped making choices consistent with their life values and goals. During MBII, many participants commented on regaining control of life, which facilitated a transition from helpless and passiveness to senses of perceived control and self‐efficacy. This theme included the following sub‐themes.

#### 3.3.1 **Both body and mind awakened**

Twenty‐seven women reported that mindfulness meditation could awaken their body and mind, making them deliberately engaging in nourishing activities rather than immersing into low mood and helplessness:

I practice body scan in the morning, which can awaken my body and mind. I feel my body cell reviving slowly, then my struggle to go to hospital and anxiety move away from my life, meanwhile my inner power is restored bit by bit. That can alleviate my physical discomfort and low mood, as if my body was purified. That was what I felt at that moment. (P5/9/13–15)

#### 3.3.2 Increasing self‐efficacy to deal with infertility and IVF‐ET

In addition, 20 participants described a new approach of taking time for themselves away from their patients’ roles, such as

doing the body scan, concentrating on the breathing, doing the exercises. That is the resource to deal with my infertility and IVF‐ET. I have more confidence and less helplessness. So I am very hopeful for the future instead of thinking that life is just going to tail off into desperation. (P10/20/2–3)

### 3.4 Increased acceptance

This theme describes that participants were better able to accommodate ongoing infertility and IVF‐related negative thoughts and emotions, having more compassion for themselves and others.

#### 3.4.1 Destigmatization

Prior to MBII, most participants described negative experiences of being misunderstood, judged and stigmatized by self and others for their infertility and IVF. The programme liberated them from the stigmatizing and self‐stigmatizing processes, developing a more positive self‐image and social identity. Thus, they learned to accept the reality of infertility and the unpredictability of IVF treatment outcome. One participant said as follows:

Initially, I can not accept my infertility because that would mean accepting I would never have a baby of my own, which makes me stigmatized. Now, I will do my best and accept the uncontrollability of IVF‐ET treatment and my imperfection. I can also accept my parents’ worry about me. Accepting my infertility does not mean giving up my journey towards parenthood, but is rather a way to create space for me to think and feel my thoughts and emotions without having to resist and avoid them. (P20/35/22)

In addition, 38 participants described the group as a place of care and support in the diaries. This was rooted in a strong sense of shared identity, which facilitated greater self‐acceptance and revised negative identities:

I enjoyed the class. Together with teachers and classmates, we learned mindfulness meditation, and discussed the difficulties and pressures that we face during IVF. I have a profound understanding because we all share a similar experience. So, during and after every class, I feel so relaxed. Good luck to all of us! (P37/68/10–12)

#### 3.4.2 Infertility and IVF‐related thoughts and emotions objectified

Twenty‐nine participants described a new perspective on their infertility and IVF‐related thoughts and feelings, for example “These thoughts and feelings do not represent myself” or “Thoughts are not facts.” Thus, infertility and IVF‐related thoughts and feelings became more acceptable:

It was really important to realize that your thoughts are not necessarily a reflection of who you are. I think that is really helpful. (P16/27/14–16)

In addition, 30 participants spoke of increased acceptance to infertility and IVF‐related negative thoughts and feelings by realizing that they will pass:

You can just go along with the fact that you do feel bad, but it is not going to last forever. It is not the end of the world. I have learned to look at my emotions as a bystander. When I was very anxious for my treatment, I jumped out of myself and looked at me from the view of a bystander. (P20/37/6–7)

#### 3.4.3 More kind and compassionate to themselves and others

Thirty‐three participants said they were less judgemental and more kind and compassionate to themselves coming from acceptance. Just as one participant wrote as follows:

I have more self‐compassion. Even if I can not do well, I would love, not punish myself. Accepting myself, the nervous and anxious self! I know that negative emotions will adversely affect fertility, but I can not control them. Please do not criticize myself! Please tolerate, embrace and love myself! (P20/36/20–21)

They also reported more tolerance, patience and empathy to others in the interviews and diaries, for example one participant said as follows:

It becomes tolerable to queue for seeing the doctor, because I have a deep empathy for other people and medical staff. (P34/8/6)

**Figure 1:**
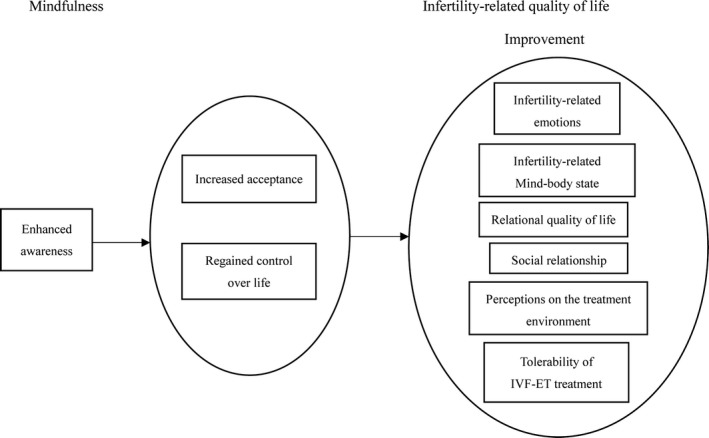
Relationship model between the themes of participants’ experiences with mindfulness

Therefore, based on the above results, a theory model on the underlying mechanism of effects of MBII on infertility‐related QoL could be developed as illustrated in Figure 1. Enhanced awareness, regained control over life and increased acceptance may be implicated in the effectiveness of MBII on the infertility‐related QoL, which could be further investigated in the future research.

## 4 DISCUSSION

These qualitative findings elucidated women’ experiences of the MBII programme and its perceived benefits of improving infertility‐related QoL, enhancing awareness, increasing acceptance and regaining control over life. This study has maximized the quantitative data obtained by contributing the subjective voices of participants and suggests that MBII may feasibly be implemented with women subjected to first IVF‐ET. Additionally, enhanced awareness, regained control over life and increased acceptance may be implicated in the effectiveness of MBII on the infertility‐related QoL.

The MBII emphasized “letting go” and encouraged a non‐judgemental perspective towards emotions, facilitating emotional acceptance in the infertile women. Mindfulness training provided a way to cultivate emotional balance (Kabat‐Zinn, 1990), and may decrease emotional reactivity, facilitating a return to baseline after reactivity (Baer, 2003). Consistent with those assumptions, in this study, participants also commented on the usefulness of MBII for alleviating operation pain and improving quality of sleep. Indeed, one of the first successful clinical applications of mindfulness was in the context of chronic pain (Kabat‐Zinn, 2013). The study by Morone et al. (2008) also demonstrated that older adults with chronic pain reported pain reduction and improved quality of sleep resulting from mindfulness meditation. They suggested that by “uncoupling” the physical sensation from the emotional and cognitive experience of pain, the patient is able to reduce pain (Morone et al., 2008).

Additionally, in the present study, most participants described greater emotional closeness with partners and friends, more satisfaction with the medical staff and services, as well as better tolerability for IVF. Social perception processes and felt connection are themselves important outcomes of mindfulness (Brown & Ryan, 2003; Carson, Carson, Gil, & Baucom, 2004; Tipsord, 2009). Previous researches (Barnes, Brown, Krusemark, Campbell, & Rogge, 2007; Burpee, & Langer, 2005; Wachs, & Cordova, 2007) indicated the positive implications mindfulness has for romantic relationship health. The breast cancer patients attending to MBSR also reported improved communication and personal relationships (Hoffman, Ersser, & Hopkinson, 2012). Mindfulness may change the perception of the self such that one begins to view the world from a broader, more objective perspective rather than a narrowly focused, self‐centred perspective (Tipsord, 2009), moving from feelings of separateness to feelings of connection (Kabat‐Zinn, 2013). Moreover, in this study, participating in MBII liberated subjects from the stigmatizing and self‐stigmatizing processes, motivating them to become more socially active. Lastly, this knowledge of the impermanence of all mental phenomena allows a higher level of tolerance of unpleasant internal states (Shapiro, Carlson, Astin, & Freedman, 2006).

Moreover, in the present study, many participants reported enhanced awareness of their bodies, thoughts and emotions, living in the moment often, and greater concentration. This finding is consistent with other research. A study on lung cancer patients participating in MBSR found that participants noticed aspects they had not been aware of before, with a greater awareness of their thoughts, emotions and physical sensations of the present moment (van den Hurk, Schellekens, Molema, Speckens, & van der Drift, 2015). A mindful, non‐judgemental experience of bodily perceptions is thought to enhance connections between the body and the mind, and to promote the acceptance of body symptoms (Mehling et al., 2011). Learning to live in the present moment is seen as a way of letting go of anxiety and re‐discovering joy (Finucane & Mercer, 2006). Through mindfulness practice, giving time to be objectively aware of the experience of life as well as living it moment to moment allowed increased acceptance, calm, confidence and ability to cope (Hoffman et al., 2012), all valuable for women facing an uncertain IVF treatment outcomes. Additionally, through mindfulness practice, participants became more conscious of their internal and experience in the present moment with an attitude of openness and curiosity. Thus, painful thoughts and feelings related to the past (e.g. ‘“previous abortion”’) or to the future (e.g. ‘“I will never be a mother”’) are recognized without trying to suppress or modify them (Galhardo et al., 2013). In addition, other study also found the significant improvements in sustained attention (Chambers, Lo, & Allen, 2008) following mindfulness meditation.

At post‐MBII, participants related to infertility and IVF‐ET in different ways, accepting them with non‐judgement and non‐reactivity, and regaining control over life. Regaining control over life is a crucial mechanism that may contribute to the changes in psychological and physical health found in MBSR interventions (Shapiro, Schwartz, & Bonner, 1998). Nearly all of the mindfulness‐based treatment programmes include acceptance of thoughts, feelings, urges, or other bodily, cognitive, and emotional phenomena, without trying to change, escape, or avoid them (Baer, 2003). Previous research also showed that breast cancer participants in the MBSR reported accepting things as they were, being less judgemental of themselves and others (Hoffman et al., 2012). In addition, in this study, the process of group members sharing experiences and developing group cohesions may enhance their acceptance of diagnosis and IVF‐ET.

As two elements of mindfulness, awareness and non‐judgemental acceptance of one's moment‐to‐moment experience are regarded as potentially effective antidotes against common forms of psychological distress (Hayes & Feldman, 2004; Kabat‐Zinn, 2013). The clinical individuals who practice these skills may experience reductions in a variety of symptom (Baer, 2003; Hofmann, Sawyer, Witt, & Oh, 2010) and improvement in QoL (Van Dam, Sheppard, Forsyth, & Earleywine, 2011). Meanwhile, by directing their attention to the “here and now,” practitioners are able to let go of fears regarding the future or ruminations about the past (Shapiro, Astin, Bishop, & Cordova, 2005; van den Hurk et al., 2015). In this way, the women learn to see their habitual reactions to infertility and IVF‐ET treatment stressors and to cultivate healthier, more adaptive ways of responding to them. Moreover, intentionally cultivating non‐judgemental attention leads to connection, which leads to greater order and health (Shapiro et al., 2006). Lastly, all patients considered the group‐interaction valuable, reducing loneliness, ameliorating stigmata, improving communication and empathy, increasing self‐efficacy.

There were some limitations in the present study. Firstly, the semi‐structured interview guide was based upon the FertiQoL survey. The use of the FertiQol could have limited the responses to the key concepts all the categories emerged from the narrative of the participants. Secondly, it is inherent in the qualitative methodology, that is the findings cannot be generalized beyond this group of participants. This study is one of possibility, rather than probability (Mackenzie, Carlson, Munoz, & Speca, 2007). Thirdly, results only reflect experiences during and immediately after the mindfulness programme and not long‐term experiences. Lastly, it is the inherently restrictive format of the diaries themselves. Certain processes of reperception may not have been captured since there were specific guidelines in the diaries (Kerr, Josyula, & Littenberg, 2011). Therefore, more methods need to be used for data collection in the study in future, such as Focus Group Discussion (FGD).

## 5 CONCLUSION

Conclusively, this qualitative assessment added to the understanding of the potential benefits of the MBII programme. The MBII has promising potential as an adjunct treatment for infertile women undergoing first IVF‐ET. Clinicians and nurses working with women undergoing IVF‐ET treatment have another tool to recommend to the patients. Furthermore, in this study, exploring IVF‐ET women's lived experience of mindfulness meditation and how they have integrated the practices into their treatment and their lives provides useful information that strongly suggests future directions for research. Lastly, according to the findings, awareness, control over life and acceptance may be potential mechanisms that lead to improved infertility‐related QoL in women following MBII programme. Therefore, that will be useful for isolating important principles of adaptive behavioural and psychological change that is helpful for perfecting the MBII techniques, ultimately improving the treatment effectiveness.

## CONFLICT OF INTEREST

The authors declare that they have no conflict of interest.
